# Role of Multiple Vitamin D-Related Polymorphisms in Multiple Sclerosis Severity: Preliminary Findings

**DOI:** 10.3390/genes13081307

**Published:** 2022-07-22

**Authors:** Luisa Agnello, Concetta Scazzone, Bruna Lo Sasso, Matteo Vidali, Rosaria Vincenza Giglio, Anna Maria Ciaccio, Paolo Ragonese, Giuseppe Salemi, Marcello Ciaccio

**Affiliations:** 1 Institute of Clinical Biochemistry, Clinical Molecular Medicine and Clinical Laboratory Medicine, Department of Biomedicine, Neurosciences and Advanced Diagnostics, University of Palermo, 90127 Palermo, Italy; luisa.agnello@unipa.it (L.A.); concetta.scazzone@unipa.it (C.S.); bruna.losasso@unipa.it (B.L.S.); rosariavincenza.giglio@unipa.it (R.V.G.); 2 Department of Laboratory Medicine, University Hospital “P. Giaccone”, 90127 Palermo, Italy; 3 Foundation IRCCS Ca’ Granda Ospedale Maggiore Policlinico, 20122 Milan, Italy; matteo.vidali@gmail.com; 4 Unit of Clinical Biochemistry, University Hospital “P. Giaccone”, 90127 Palermo, Italy; annamaria.ciaccio@unipa.it; 5 Unit of Neurology, Department of Biomedicine, Neurosciences and Advanced Diagnostics, University of Palermo, 90127 Palermo, Italy; paolo.ragonese@unipa.it (P.R.); giuseppe.salemi@unipa.it (G.S.)

**Keywords:** genetic, prognosis, severity, SNP, MS

## Abstract

Background: Multiple Sclerosis (MS) is a multifactorial disease whose pathogenesis is the result of interaction among genetic, epigenetic, and environmental factors. Among these, a role for vitamin D hypovitaminosis has emerged in recent decades. Vitamin D levels are influenced by both environmental and genetic factors. Single nucleotide polymorphisms (SNPs) in genes codifying for molecules involved in vitamin D metabolism have been associated with an increased risk of developing MS. However, few studies assessed the association of such SNPs with the severity of the disease. The aim of this observational study was to evaluate the potential association among vitamin D status, MS severity, and vitamin D-related SNPs, alone or in combination. Methods: In a cohort of 100 MS patients, we genotyped 18 SNPs in the following genes: NAD synthetase 1, CYP2R1, vitamin D binding protein, vitamin D receptor, Retinoid X Receptor-α, KLOTHO, CYP24A1, and CYP27A1. Serum 25(OH)D3 levels were measured by high-performance liquid chromatography. Genotyping was performed by real-time polymerase chain reaction or PCR-RFLP. Results: We did not find any association between SNPs, alone or in combination, and MS severity. Conclusion: In this study, we make an initial evaluation of the possible influence of several SNPs in vitamin D-related genes on MS severity.

## 1. Introduction

Multiple sclerosis (MS) is a chronic inflammatory autoimmune disease of the central nervous system, characterised by focal lesions of primary demyelination and neurodegeneration. It is a multifactorial disease whose pathogenesis could be a result of the interaction among environmental, genetic, and epigenetic factors. Among these, a role for vitamin D has been proposed.

Vitamin D is a secosteroid, which plays pleiotropic functions. Beyond its well-known role in calcium homeostasis, it is an important regulator of the immune system [1,2]. Accordingly, low vitamin D levels have been involved in the pathogenesis of several autoimmune diseases, including MS. A relationship between hypovitaminosis D and MS susceptibility has been hypothesised since the late nineties. In recent decades, several observational studies worldwide showed an inverse association between vitamin D levels and MS risk [3,4]. Notably, vitamin D levels are influenced by both environmental and genetic factors. Accordingly, the potential role of single nucleotide polymorphisms (SNPs) in genes codifying molecules involved in vitamin D metabolism in MS susceptibility has also been evaluated, achieving contrasting results [5]. Less investigated is the role of such SNPs in the MS severity.

In this observational study, we evaluated the potential association among vitamin D-related SNPs, vitamin D status, and MS severity. Specifically, we evaluated 18 SNPs in the following genes: NAD synthetase 1 (NADSYN1), CYP2R1, vitamin D binding protein (VDBP), vitamin D receptor (VDR), Retinoid X Receptor-α (RXR- α), KLOTHO, CYP24A1, and CYP27A1 (Table 1).

## 2. Material and Methods

### 2.1. Study Population

This was an observational, retrospective study performed at the University of Palermo, Italy.

Patients with MS were enrolled at the Unit of Neurology, Department of Biomedicine, Neurosciences, and Advanced Diagnostics.

Diagnosis of MS was made by an experienced neurologist and based on a previous history of the disease, physical examination, cerebrospinal fluid analysis, and magnetic resonance imaging findings, according to revised McDonald criteria [6]. The neurological status of patients was assessed using Kurtzke’s Expanded Disability Status Scale (EDSS). The progression of disability was assessed using the Multiple Sclerosis Severity Score (MSSS) [7]. The annualized relapse rate (ARR) was calculated in the year prior to the genotyping. At enrolment, all patients did not have clinical or radiological relapse or remission and had not been subject to corticosteroid treatment for at least one month. Patients were excluded if they did not provide informed consent or had other autoimmune diseases.

### 2.2. Biochemical and Genetic Analyses

All laboratory analyses, including vitamin D status evaluation and genotyping, were performed at the Institute of Clinical Biochemistry, Clinical Molecular Medicine and Laboratory Medicine, Department of Biomedicine, Neurosciences, and Advanced Diagnostics.

We selected 18 SNPs in genes codifying for molecules involved in the metabolism of vitamin D. The characteristics of all SNPs investigated are summarised in Table 1.

SNPs in *NADSYN1*, *CYP2R1*, *CYP24A1*, *CYP27A1*, *RXR-α*, and *KLOTHO* genes were analysed by real-time allelic discrimination TaqMan assay on a 7500 real-time polymerase chain reaction (PCR) system (Applied Biosystems, Monza, Italy), as previously described [8,9,10,11]. SNPs in *VDR* and *VDBP* genes were assessed by PCR followed by restriction fragment length polymorphism (RFLP) analysis, as previously described [12,13]. Vitamin D status was evaluated by measuring serum 25-hydroxy-vitamin D3 [25(OH)D_3_] levels using high-performance liquid chromatography (HPLC).

### 2.3. Statistical Analysis

Statistical analysis was performed by SPSS version 17.0 (SPSS Inc., Chicago, IL, USA) and R Language v.3.6.1 (R Foundation for Statistical Computing, Vienna, Austria). Quantitative variables were expressed by the median and interquartile range (IQR), while categorical variables by relative frequencies. Association between polymorphisms was evaluated by Fisher’s Exact test. Univariate analyses to evaluate the association between MSSS and vitamin D levels were performed by Kruskal–Wallis and General Linear Model (GLM). The analysis was also separately conducted on RRMS subgroup only. Multivariate analysis was performed by GLM, using a continuous dependent variable (MSSS or vitamin D) and binary predictors (form of the disease and/or polymorphisms). A new variable called “number of polymorphisms” was created, using, for each polymorphism, 0 as WT and 1 as the presence of polymorphism. Correlation between number of polymorphisms (n) and MSSS, or vitamin D, was evaluated by Spearman’s rank correlation. Considering that the IQR of the new variable was 10–13, we have arbitrarily selected the two groups with the most extreme values, n ≤ 9 and n ≥ 14 polymorphisms (about 15% of patients in each group). These groups were compared for difference in MSSS score or vitamin D levels by the non-parametric Mann–Whitney test.

## 3. Results

We included a total of 100 MS patients, of whom 82 had RRMS form and 14 SPMS form. Demographics, clinical and biochemical characteristics, as well as allele frequencies of studied polymorphisms, are shown in Table 2. At the univariate analysis, the association between dependent variables (MSSS or vitamin D) and predictors (demographics, clinical variables, and genotypes) was further evaluated. Form of the disease (*p* = 0.002) and *CYP24A1* rs2762939 were found to be significantly associated with MSSS (Table 3); whereas all *NADSYN1* gene polymorphisms (*p* values ranging from 0.024 to 0.047) and *CYP24A1* rs2248137 (*p* = 0.026) were found to be significantly associated with vitamin D levels (Table 3). Post-hoc comparisons (between homozygosis for the ancestral gene, heterozygosis, and homozygosis for the substitution gene) were not statistically significant, if taking into account Bonferroni’s correction.

Additionally, we evaluated the associations between polymorphisms and vitamin D in the RRMS subgroup. However, in this subgroup no polymorphism resulted to be associated with vitamin D levels. Polymorphisms found to be associated in the whole sample in the RRMS subset were found to be closed to statistical significance but with *p* > 0.05 (0.06–0.1). This discrepancy between significances found in the subset and in the whole sample is possible due to reduced sample size, hence reduced statistical power.

Multivariate analysis was performed considering only variables found to be associated at the univariate analysis. To this aim, MSSS was considered a continuous dependent variable, while form of the disease (RRMS vs. all others) and the *CYP24A1* rs2762939 gene polymorphism (GG + GC vs. CC) were taken as binary independent variables. The analysis showed that the form of the disease (*p* = 0.006), but not the *CYP24A1* rs2762939 gene polymorphism (*p* = 0.072), is an independent predictor of MSSS (adjusted R squared for the model 0.70). Same results were obtained considering the GG vs. GC + CC for the *CYP24A1* rs2762939 gene polymorphism.

Multivariate analysis for vitamin D levels was also conducted. Since *NADSYN1* rs3829251 and rs7944926 were significantly associated (*p* < 0.001), only *NADSYN1* rs3829251 and rs12785878 and *CYP24A1* rs2248137 were included in the multivariate model. Only *NADSYN1* rs3829251 was found to be an independent predictor of vitamin D levels (*p* = 0.025), while *NADSYN1* rs12785878 (*p* = 0.786) or *CYP24A1* rs2248137 (*p* = 0.387) were not associated.

We further performed sub-analysis recoding allele variables (3 levels: homozygosis for the ancestral gene (0), heterozygosis (1), homozygosis for the substitution gene (2)) into binary variables: in the 1st sub-analysis 0 + 1 vs. 2; in the 2nd sub-analysis 0 vs. 1 + 2. For all polymorphisms investigated, the groups were compared for EDSS, MSSS, and ARR (only comparisons with a minimum sample size for each group of 5 patients were considered). In the first sub-analysis, no association was found; in the second sub-analysis an association was found between EDSS and *CYP24A1* rs2762939 (*p* = 0.024)

A score summing up all polymorphisms (using 0 as WT and 1 as the presence of polymorphism) was calculated. The median (IQR, min–max) number of polymorphisms was 11 (10–13, 7–16). Number of polymorphisms did not correlate with MSSS (rho = 0.051, *p* = 0.676) or with vitamin D levels (rho = −0.046, *p* = 0.681). Accordingly, patients with more polymorphisms (n ≥ 14) did not show significantly higher MSSS or vitamin D levels than patients with fewer polymorphisms (n ≤ 9), with *p* = 0.526 and *p* = 0.917, respectively.

## 4. Discussion

In this study, we assessed the hypothesis that the presence of vitamin D-related SNPs could influence MS severity. The main finding of our study can be summarised as follows: (i) the form of the disease is an independent predictor of MSSS, as expected; (ii) among all investigated SNPs, only the *CYP24A1* rs2762939 is significantly associated with MSSS; (iii) all SNPs of *NADSYN1* and rs2248137 of *CYP24A1* are associated with decreased vitamin D levels, with rs3829251 being independently associated at the multivariate analysis; (iv) the simultaneous presence of multiple SNPs is not associated with the disease severity. To the best of our knowledge, this is the first study that evaluates the cumulative effect of vitamin D-related SNPs on MS severity. Indeed, each patient enrolled harboured at least seven SNPs. Specifically, we selected SNPs in genes codifying for molecules with an important role in the vitamin D pathway. The *NADSYN1* gene codifies for an enzyme that catalyses NAD synthesis, a coenzyme involved in 25(OH)D synthesis and hydroxylation. CYP2R1 and CYP27A1 catalyse both the reaction of 25-hydroxylation of vitamin D, leading to the production of 25(OH)D. CYP2R1 is located in the liver and represents the major contributor to vitamin D 25-hydroxylation. Also, CYP27A1 is a hepatic enzyme and participates to the hydroxylation of vitamin D but to a lesser extends than CYP2R1. VDBP is fundamental for vitamin D and related metabolites transport in the circulation and, consequently, regulates vitamin D availability to target cells [14]. Vitamin D exerts its biological function through the interaction with the intracellular heterodimer complex consisting of VDR and RXR-α. After binding the active form of vitamin D, namely 1-α 25-dihydroxyvitamin D (1α,25(OH)_2_D), the complex migrates to the nucleus where it interacts with Vitamin D Responsive Element (VDRE) located in the promoter region of several genes. Finally, Klotho is an important regulator of vitamin D homeostasis. We chose to evaluate SNPs in the above-mentioned genes because they were previously associated with altered vitamin D status. Although the investigated SNPs have been associated with MS susceptibility [8,10,12,13,15,16,17], they do not seem to affect disease severity, except for *CYP24A1* rs2762939, according to our findings. This is the first study revealing a possible role of *CYP24A1* genetic alteration in MS severity. Recently, Malhotra et al. showed that another SNP in the *CYP24A1* gene, namely the rs2762943, is associated with MS susceptibility but not with severity [18].

Overall, most studies failed to find an association between polymorphisms in several genes, including vitamin D-related ones, and MS severity [19,20,21,22,23]. Thus, the contribution of genetics to MS severity remains elusive.

It can be postulated that mechanisms that predispose individuals to develop MS could diverge from those that drive disease progression. The contribution of genetics to MS severity is still far from being elucidated. Indeed, several mechanisms could be involved in the disease progression, including the effect of treatments, environment, and stochastic processes. It is plausible that common genetic variants exert only a weak influence on disease progression.

Our study has some limitations. We included a small number of MS patients, and we did not perform a longitudinal evaluation. Moreover, since for some polymorphisms very few subjects displayed the ancestral or the substitution allele, a lack of association with MSSS or vitamin D could be possibly due to the limited statistical power. The strength is that we evaluated 18 vitamin D-related SNPs for each patient.

In conclusion, in this study, we first evaluate the possible influence of several SNPs in vitamin D-related genes on MS severity. Although we did not find any association between investigated SNPs and MS progression, further studies are required to confirm such findings.

## Figures and Tables

**Table 1:** Characteristics of vitamin D-related SNPs.

Gene	Chromosome	SNP	Ancestral Allele	Substitution Allele
*NADSYN1*	11	rs3829251	G	A
		rs7944926	G	A
		rs12785878	G	T
*CYP2R1*	11	rs10766197	G	A
		rs10741657	G	A
*VDBP*	4	rs7041	G	T
		rs4588	C	A
*VDR*	12	rs1544410 (Bms-I)	B	b
		rs7975232 (Apa-I)	A	a
		rs731236 (Taq-I)	T	t
		rs2228570 (Fok-I)	F	f
*RXR-α*	9	rs9409929	G	A
		rs12004589	G	A
*KLOTHO*	13	rs9536314	T	G
		rs1207568	G	A
*CYP24A1*	20	rs2762939	G	C
		rs2248137	G	C
*CYP27A1*	2	rs17470271	T	A

NADSYN1, NAD Synthetase 1; VDBP, Vitamin D Binding Protein; VDR, Vitamin D Receptor; RXR-α, Retinoid X Receptor-α; ICV, Initial Codon Variant.

**Table 2:** Characteristics of the study population (n = 100).

Variable	
Demographics	
Sex, (%)	M: 25% F: 75%
Age, years (median, IQR)	39 (32–47)
Clinical	
Form of the disease, (%)	RRMS: 82% SPMS: 14% Other: 4%
EDSS, (median, IQR)	2.3 (1.4–5.0)
MSSS, (median, IQR)	3.34 (1.45–5.48)
ARR, %	0: 22% 1: 41% 2: 29% 3: 5% 4: 3%
Genotype	
*NADSYN1* rs3829251, (%)	GG 75%, GA 25%
*NADSYN1* rs7944926, (%)	GG 43%, GA 52%, AA 5%
*NADSYN1* rs12785878, (%)	GG 5%, GT 51%, TT 44%
*CYP2R1* rs10766197, (%)	GG 19%, GA 44%, AA 37%
*CYP2R1* rs10741657, (%)	GG 59%, GA 4%, AA 37%
*VDBP* rs7041, (%)	GG 38%, GT 43%, TT 19%
*VDBP* rs4588, (%)	CC 63%, CA 31%, AA 6%
*VDR* FOK-I, (%)	FF 34%, Ff 47%, ff 19%
*VDR* BSM-I, (%)	BB 22%, Bb 48%, bb 30%
*VDR* APA-I, (%)	AA 29%, Aa 55%, aa 16%
*VDR* TAQ-I, (%)	TT 33%, Tt 47%, tt 20%
*RXR-α* rs9409929, (%)	GG 48% GA 44% AA 8%
*RXR-α* rs12004589, (%)	GG 80%, GT 20%
*KLOTHO* rs9536314, (%)	TT 75%, TG 23%, GG 2%
*KLOTHO* rs1207568, (%)	GG 69%, GA 28%, AA 3%
*CYP24A1* rs2762939, (%)	GG 52%, GC 42%, CC 6%
*CYP24A1* rs2248137, (%)	GG 17%, GC 45%, CC 38%
*CYP27A1* rs17470271, (%)	TT 12%, TA 51%, AA 37%
Biochemical	
Vitamin D, μg/L (median, IQR)	20 (16–25)

RRMS, Relapsing-Remitting Multiple Sclerosis; SPMS, Secondary progressive Multiple Sclerosis; EDSS, Expanded Disability Status Scale; MSSS, Multiple Sclerosis Severity Score; ARR, Annualized Relapse Rate; NADSYN1, NAD Synthetase 1; VDBP, Vitamin D Binding Protein; VDR, Vitamin D Receptor; RXR- α, Retinoid X Receptor-α.

**Table 3:** Univariate analysis to predict MSSS and vitamin D levels.

Independent Variable	MSSS	Vitamin D
Sex	*p* = 0.133	*p* = 0.604
Age	*p* = 0.764	*p* = 0.500
Form of the disease (RRMS vs. all others)	*p* = 0.002 * RRMS 2.44 Others 6.47	*p* = 0.294
MSSS	-	*p* = 0.146
*NADSYN1* rs3829251	*p* = 0.860	*p* = 0.024 * GG 22 GA 19
*NADSYN1* rs7944926	*p* = 0.816	*p* = 0.025 * GG 24 GA 19 AA 24
*NADSYN1* rs12785878	*p* = 0.920	*p* = 0.047 * GG 24 GT 19 TT 24
*CYP2R1* rs10766197	*p* = 0.888	*p* = 0.771
*CYP2R1* rs10741657	*p* = 0.667	*p* = 0.732
*VDBP* rs7041	*p* = 0.952	*p* = 0.954
*VDBP* rs4588	*p* = 0.651	*p* = 0.310
*VDR* FOK-I	*p* = 0.461	*p* = 0.397
*VDR* BSM-I	*p* = 0.632	*p* = 0.322
*VDR* APA-I	*p* = 0.297	*p* = 0.992
*VDR* TAQ-I	*p* = 0.192	*p* = 0.185
*RXR-α* rs9409929	*p* = 0.404	*p* = 0.086
*RXR-α* rs12004589	*p* = 0.085	*p* = 0.392
*KLOTHO* rs9536314	*p* = 0.412	*p* = 0.590
*KLOTHO* rs1207568	*p* = 0.170	*p* = 0.171
*CYP24A1* rs2762939	*p* = 0.042 * GG 4.55 GC 2.44 CC 0.53 GG vs. CC *p* = 0.041 (not significant if considering Bonferroni’s correction)	*p* = 0.987
*CYP24A1* rs2248137	*p* = 0.763	*p* = 0.026 * GG 20 GC 24 CC 19
*CYP27A1* rs17470271	*p* = 0.061	*p* = 0.607

NADSYN1, NAD Synthetase 1; VDBP, Vitamin D Binding Protein; VDR, Vitamin D Receptor; RXR-α, Retinoid X Receptor-α; ICV, Initial Codon Variant. * *p* statistically significant.

## Data Availability

The data that support the findings of this study are available from the corresponding author upon reasonable request.
